# Effects of Gold Salt Speciation and Structure of Human and Bovine Serum Albumins on the Synthesis and Stability of Gold Nanostructures

**DOI:** 10.3389/fchem.2016.00013

**Published:** 2016-03-31

**Authors:** Érica G. A. Miranda, Aryane Tofanello, Adrianne M. M. Brito, David M. Lopes, Lindomar J. C. Albuquerque, Carlos E. de Castro, Fanny N. Costa, Fernando C. Giacomelli, Fabio F. Ferreira, Juliana C. Araújo-Chaves, Iseli L. Nantes

**Affiliations:** Centro de Ciências Naturais e Humanas, Universidade Federal do ABCSanto André, Brazil

**Keywords:** human serum albumin, bovine serum albumin, gold nanoparticles, pH, gold speciation, protein structure, reducing and stabilizing agents

## Abstract

The present study aimed to investigate the influence of albumin structure and gold speciation on the synthesis of gold nanoparticles (GNPs). The strategy of synthesis was the addition of HAuCl_4_ solutions at different pH values (3–12) to solutions of human and bovine serum albumins (HSA and BSA) at the same corresponding pH values. Different pH values influence the GNP synthesis due to gold speciation. Besides the inherent effect of pH on the native structure of albumins, the use *N*-ethylmaleimide (NEM)-treated and heat-denaturated forms of HSA and BSA provided additional insights about the influence of protein structure, net charge, and thiol group approachability on the GNP synthesis. NEM treatment, heating, and the extreme values of pH promoted loss of the native albumin structure. The formation of GNPs indicated by the appearance of surface plasmon resonance (SPR) bands became detectable from 15 days of the synthesis processes that were carried out with native, NEM-treated and heat-denaturated forms of HSA and BSA, exclusively at pH 6 and 7. After 2 months of incubation, SPR band was also detected for all synthesis carried out at pH 8.0. The mean values of the hydrodynamic radius (RH) were 24 and 34 nm for GNPs synthesized with native HSA and BSA, respectively. X-ray diffraction (XRD) revealed crystallites of 13 nm. RH, XRD, and zeta potential values were consistent with GNP capping by the albumins. However, the GNPs produced with NEM-treated and heat-denaturated albumins exhibited loss of protein capping by lowering the ionic strength. This result suggests a significant contribution of non-electrostatic interactions of albumins with the GNP surface, in these conditions. The denaturation of proteins exposes hydrophobic groups to the solvent, and these groups could interact with the gold surface. In these conditions, the thiol blockage or oxidation, the latter probably favored upon heating, impaired the formation of a stable capping by thiol coordination with the gold surface. Therefore, the cysteine side chain of albumins is important for the colloidal stabilization of GNPs rather than as the reducing agent for the synthesis. Despite the presence of more reactive gold species at more acidic pH values, i.e., below 6.0, in these conditions the loss of native albumin structure impaired GNP synthesis. Alkaline pH values (9–12) combined the unfavorable conditions of denaturated protein structure with less reactive gold species. Therefore, an optimal condition for the synthesis of GNPs using serum albumins involves more reactive gold salt species combined with a reducing and negatively charged form of the protein, all favored at pH 6–7.

## Introduction

The pioneering work of Faraday in the nineteenth century identified for the first time, the distinct properties of gold at the nanoscale. He assigned the red coloration exhibited by an HAuCl_4_ solution after the reaction with white phosphorus to the presence of colloidal gold. Since then interest in metal nanostructures has been growing, and several subsequent studies concerned with metal nanostructures were equally remarkable. The basis for subsequent advances in the domain of the production and properties of nanostructures were the solution of Maxwell's equations for spherical particles by Mie (1908) and the landmark paper of Turkevich in 1951 describing a simple and efficient synthesis method of gold nanoparticles (GNPs) (Mie, 1908; Turkevich et al., 1951; Cobley et al., 2011). In parallel, the invention of the ultramicroscope by Richard Zsigmondy was crucial for the development of nanoscience because it permitted the direct observation of the NPs. In sequence, the advances comprised the control of size and morphology of nanostructures and the discovery of new properties and applications for these materials (Schmid, 1992; Grzelczak et al., 2008). The noble metals are widely used for the construction NPs. The metal NPs exhibit exclusive photonic, electronic and catalytic properties. These properties include local plasmon resonance surface (LSPR) (Toma et al., 2010; Rodríguez-Oliveros and Sánchez-Gil, 2012), improved surface Raman scattering (SERS) (Maiorano et al., 2011), and improved surface fluorescence (SEF) (Xie et al., 2006). These specific properties come from the confinement of the electrons on the surface of the nanoscale structure. Thus, the properties of metal nanoparticles can be modulated by constructing nanostructures with different sizes and shapes. The synthesis of metal nanoparticles can occur by a physical method that consists in fractioning the bulk structure to nanometric parts or by chemical synthesis using metallic salts and a reducing agent. Nowadays, the advent of green chemistry motivated the search for non-polluting methods of one-step synthesis to the production of metallic nanoparticles (MNPs). In this regard, isolated biomolecules and their mimetics, cellular extracts, and even whole microorganisms have the necessary characteristics required for a the eco-friendly synthesis of MNPs with the additional benefits of compatibility for pharmaceutical and other biomedical applications (Nghiem et al., 2010). Also, the biological macromolecules, such as proteins, have mild reducing chemical groups and offer the advantage to act as biotemplates for directing the growing and geometry of MNPs. Therefore, biological macromolecules came under the spotlight in the development of green, and one pot methods for the synthesis of MNPs. Particularly, cysteine-containing peptides and proteins have gained attention as efficient reducing and stabilizing agents for the synthesis of MNPs (Pereira et al., 2011; Durán et al., 2015). The cysteine -SH is a powerful reducing agent and both –SH and –S-S- can contribute for MNP capping. In this regard, bovine and human serum albumins (BSA and HSA), largely used for MNP synthesis, have 17 disulfide bonds and a free thiol group provided by Cys34 (Quinlan et al., 2005). The importance of serum albumins for the synthesis of MNPs, particularly for GNP synthesis, is well evidenced in the literature. Albumin acts as stabilizing and reducing agent and can direct the one step synthesis of GNPs with different sizes and shapes. The formation of GNP using albumins can be controlled by carefully choosing the reaction conditions. Albumin can shift GNP synthesis toward different morphologies and properties by the manipulation of temperature, pH and the use of a diversity of additives (Basu et al., 2008; Focsan et al., 2011). BSA is an excellent foaming agent which, in its zwitterionic form, can bind cationic and anionic ions such as silver (Ag^+^) and gold (AuCl${}_{4}^{2-}$). This property of BSA exhibited at its isoelectric point allows the synthesis Au–Ag alloy NPs (Singh et al., 2005). The conjugation of BSA on the surface of GNPs provides differentiated properties and biocompatibility of these nanostructures and allows their use as sensor as well as in cancer therapy and drug delivery (Hu et al., 2010; Khullar et al., 2012; Mocan et al., 2015; Zelasko-Leon et al., 2015; Gopu et al., 2016). Regarding biocompatibility, in a real-serum environment, albumins followed by transferrin were observed as predominant proteins composing the corona of GNPs (Matczuk et al., 2015). The nucleation and growth of MNPs synthesized using macro-biomolecules such as albumins are modulated by the template provided by the molecular structure and the solvation (Saptarshi et al., 2013). Thus, albumins can produce spherical, multibranched and polyhedral GNPs as well as gold nanoplates. In the protocols of MNP synthesis using biomolecules such as proteins, the pH of the medium has a significant influence not only on the structure and the reducing capacity of the biomolecule but also on the speciation of aqueous HAuCl_4_ (Table 1). The increase of the pH of an HAuCl_4_ solution leads to progressive hydrolysis of the salt with repercussions on the oxidizing capacity of the species. At pH ≤ 4, Au(III) chloride exists in the planar [AuCl_4_]^−^ form and is the gold species more favorable for the reduction. At 6 < pH < 8, the predominant species are [AuCl_3_(OH)]^−^, [AuCl_2_(OH)_2_]^−^ and [AuCl(OH)_3_]^−^. Ultimately, at pH > 8 the predominant species is [Au(OH)_4_]^−^, that is the less efficient oxidant species (Moreau et al., 2005).

**Table 1:** **Speciation of Aqueous [AuCl_4_]^−^ at Various pH values**.

**pH**	**Gold ion species (%)**					
	**AuCl${}_{4}^{-}$**	**AuCl_3_ (OH)^−^**	**AuCl_3_ (H_2_O)**	**AuCl_2_ (OH)_2_**	**AuCl_2_ (OH)_3_**	**Au (OH)${}_{4}^{-}$**
1	100	0	0	0	0	0
2	90	4	6	0	0	0
3	50	20	30	0	0	0
4	8	33	47	12	0	0
5	0	12	18	52	18	0
6	0	0	1	20	75	4
7	0	0	0	2	70	28
8	0	0	0	0	20	80
9	0	0	0	0	3	97
10	0	0	0	0	0	100
11	0	0	0	0	0	100
12	0	0	0	0	0	100

According to previous data of literature, the reduction potential of the aqueous HAuCl_4_ solution measured at pH 2.91, 6.16, and 8.01 revealed reduction potentials of 0.66, 0.59, and 0.53 V, respectively. The voltammogram of the aqueous HAuCl_4_ solution at pH 10.35 did not exhibit a reduction peak (Wang et al., 2009).

In the present study, we synthesized GNPs using human and bovine serum albumins as the agents for gold biomineralization on the pH scale of 3–12. The proteins were used in the native form, unstructured by heating and with cysteine thiol groups blocked by NEM (*N*-ethylmaleimide). The synthesis processes were carried out by mixing protein and gold solutions with the same desired pH values, i.e., as an example, a protein solution at pH 8 was mixed with a gold solution at pH 8. The objective was to identify the possible critical conditions for the GNP formation and stabilization: the protein structure, the availability of the unique free thiol group of the albumins and the gold speciation.

## Materials and methods

### Chemicals

Hydrogen tetrachloroaurate (HAuCl_4_), bovine serum albumin (BSA), human serum albumin (HSA), *N*-ethylmaleimide (NEM), sodium phosphate, chloride acid, and sodium hydroxide were purchased from Sigma-Aldrich. All aqueous suspensions and solutions were prepared with deionized water (mixed bed of ion exchange, Millipore^®^), and the pH was measured using a combined glass electrode (Orion Glass pH SURE-FLOW™). The reference electrode (ROSS™, model 8102) was filled with OrionFilling Solutions (ROSS™). The pH meter was calibrated using METREPAK pHydrion standard buffer solutions (Brooklyn, NY) Chemicals. All glasswares were cleaned with an aqua regia solution (1:3–HNO_3_/HCl) for complete removal of potential artificial nucleation sites.

The 450 μM salt HAuCl_4_ were dissolved in 26 mM sodium phosphate buffer. The pH 3–12 of stock solution serum and salt HAuCl_4_ was adjusted using HCl 1M or NaCl 1M. Proteins (BSA 67 kDa and HSA 66 kDa) were dissolved in Milli-Q^®^ water to make up 30 mM protein stock solutions at pH 3–12. Aliquots were collected in each pH and separated into three aliquots of 100 μl. For the reaction with NEM, a freshly prepared stock solution of the thiol reactant prepared in deionized water was added to the albumin solution to a final concentration of 60 μM. For heat-promoted denaturation of the protein, a 100 μL of albumin solution was heated at 85°C for 30 min. HSA and BSA (native form, NEM-treated and heat-denaturated) solutions were mixed with the HAuCl_4_ solution in the presence of sodium phosphate, and the pH ascertained to the values of 3.0–12.0, with one unit of the interval. The concentration of serum albumin was maintained at 1.5 μM.

### Electronic absorption spectra measurements

The electronic absorption spectra of albumins were measured by using a Varian Cary 50, Varian Inc. (CA, United States) spectrometer. The spectral resolution for wavelength scan was 0.5 nm. The optical path length was 1 cm for the porphyrin and GNP spectra measurements.

### CD measurements

The CD measurements were carried out in a Jasco J-720 spectropolarimeter (Easton, MD) using quartz cuvettes with a 0.1 cm optical path; bandwidth, 1.0 nm; scanning speed, 100 nm/min; response, 0.5 s; and accumulations, 4.0.

### FT-raman

Raman spectra were recorded on a Bruker Optics spectrometer equipped with a diode-pumped Nd:YAG (neodymium-doped yttrium aluminum garnet) laser emitting at 1064 nm (laser power: 100 mW). The spectra were recorded at a resolution of 4 cm^−1^ accumulating 320 scans, over the wavenumber range from 70 to 4000 cm^−1^. In the case of the liquid samples, 250 μL were used for the spectroscopic measurements.

### X-ray powder diffraction (XRD)

The GNP solution was dripped onto a 0.014-mm thick cellulose acetate foil forming a thin film (dried at room temperature). Then the film was accommodated in a sample holder that was held spinning during data collection. X-ray powder diffraction data were recorded at room temperature on a STADI-P powder diffractometer (Stoe^®^, Darmstadt, Germany) in transmission geometry by using a Mo K_α1_ (λ = 0.7093 Å) wavelength selected by a curved Ge (111) crystal, with a tube voltage of 50 kV and a current of 40 mA. The diffracted intensities were recorded by a silicon microstrip detector, Mythen 1K (Dectris^®^, Baden, Switzerland), in the range from 12° to 50°, with step sizes of 0.015.

### Electrophoretic light scattering (ELS)

The ELS measurements were performed to determine the average zeta potential (ζ) of the assemblies using the Zetasizer NanoZS equipment (Malvern Instruments, UK). The equipment measures the electrophoretic mobility (U_E_) of the scattering objects and converts the value to a ζ potential (mV) through the well-known Henry's equation which has been calculated through the Smoluchowski approximation. Each ζ potential value reported herein is an average of 10 independent measurements with a repeatability of ±2%.

### Dynamic light scattering (DLS)

DLS measurements were performed using an ALV/CGS-3 compact goniometer system consisting of a 22 mW HeNe linearly polarized laser operating at a wavelength of 633 nm, an ALV 7004 digital correlator and a pair of avalanche photodiodes operating in pseudo cross-correlation mode. The samples were placed in 10 mm diameter glass cells and maintained at a constant temperature of 25 ± 1°C. The autocorrelation functions reported are based on 03 independent runs of 60 s counting time. The data were collected and further averaged by using the ALV Correlator Control software. The averaged intensity correlation functions *g*_2_(*t*) were analyzed using the algorithm REPES (Jakes, 1995; incorporated in the GENDIST program), resulting in distributions of relaxation times *- A*(τ). Wherever noticed, the distributions of relaxation times were also converted to distributions of R_H_ by using the Stokes-Einstein equation. The polydispersity of the nanoparticles was accessed by using the cumulant analysis (Stepánek, 1993) of the autocorrelation functions measured at 90°.

## Results

**Figure 1:**
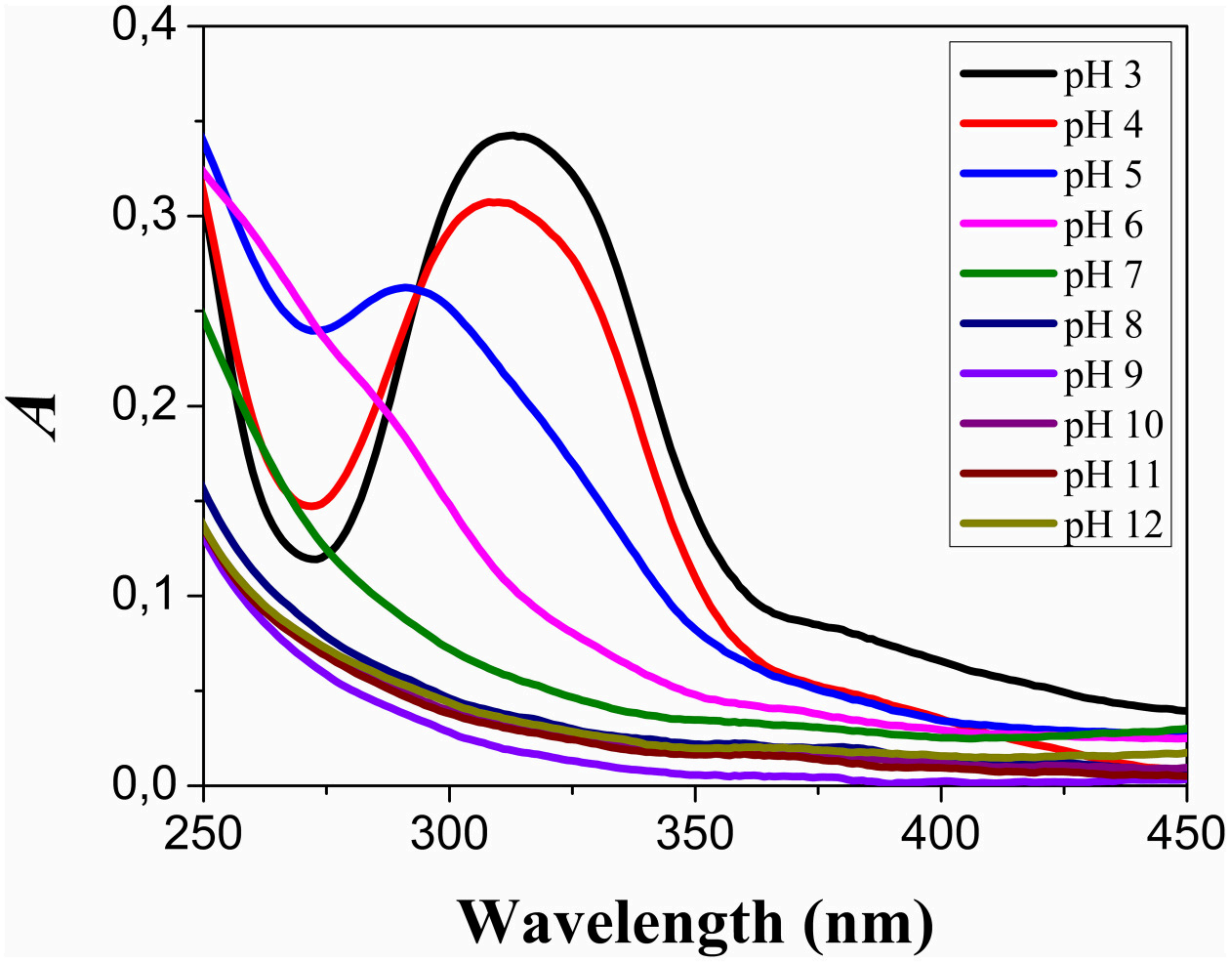
**pH-dependent speciation of HAuCl_4_**. The electronic absorption spectra of the different species of gold salt were obtained with 0.45 mM HAuCl_4_ solutions in the presence sodium 26 mM of sodium phosphate, at the pH range of 3–12.

The reduction potential of aqueous solutions of HAuCl_4_ is pH-dependent. Accordingly to previous data of literature, the reduction potential of aqueous HAuCl_4_ measured at pH 2.91, 6.16, and 8.01 revealed reduction potentials of 0.66, 0.59, and 0.53 V, respectively. The voltammogram of aqueous HAuCl_4_, at pH 10.35, did not exhibit a reduction peak (Wang et al., 2009). These results indicated that the species [AuCl4]^−^, [AuCl_3_(OH)]^−^, [AuCl(OH)_3_]^−^, and [Au(OH)_4_]^−^ are in a decreasing order of reactivity to produce gold colloids. Figure 1 shows the UV-visible spectra of HAuCl_4_ solutions at the pH range of 3–12 with an interval of 1 unit of pH.

**Figure 2:**
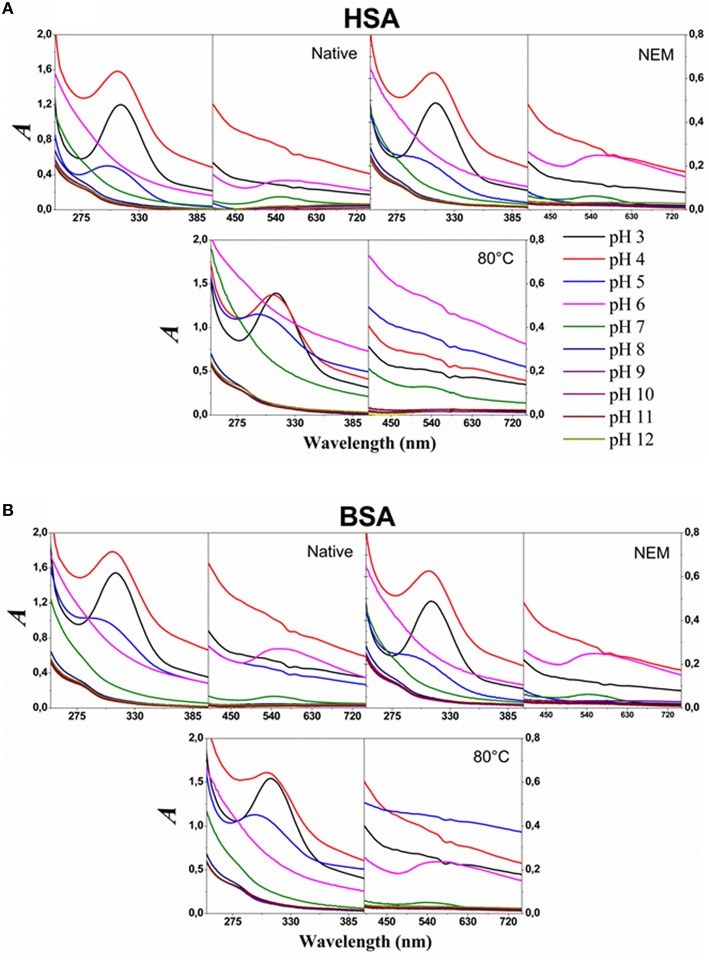
**Electronic absorption spectra of the product of the reactions after 15 days of incubation**. The upper and lower panels show the spectra of HSA **(A)** and BSA **(B)** solutions after 15 days of incubation, at room temperature, with HAuCl_4_ solutions at the different indicated pH values as indicated in the figure. A solution of 0.45 mM solution of HAuCl_4_ was added to 1.5 μM solution of the proteins in the conditions indicated in the panel. The solutions contained 26 mM of sodium phosphate buffer.

The spectra shown in Figure 1 are similar to the results previously presented in the literature and consistent with the speciation of aqueous HAuCl_4_ solutions. The UV-visible absorption spectra obtained at pH 3 presented a band with a peak at 312 nm. This band is a composite spectrum that could be assigned to two unresolved ligands (π) to metal (σ^*^) charge transfer (LMCT) bands (Wang et al., 2009). The peak of this band was blue shifted to 310 nm at pH 4.0, and 293 nm at pH 5.0. In this condition, the blue shift was accompanied by a decrease in the absorption intensity. The LMCT band was not detected at pH ≥ 6.0. The HAuCl_4_ solutions at different pH values were added to respective HSA and BSA solutions titrated to the same corresponding pH values. The final concentration of the albumins was 1.5 μM and of the HAuCl_4_ was 0.45 mM. HSA and BSA were in three different conditions: native form, heated at 85°C and treated with NEM. The use of native and heat-denaturated forms of HSA and BSA had the objective to investigate the influence of the protein structure and thiol group approachability on the GNP synthesis. The use of albumins previously treated with NEM had the objective to investigate the importance of the thiol group of the albumins for the synthesis and stabilization of GNPs. The samples containing HAuCl_4_ and albumins were incubated in the dark at room temperature, and the solution color checked daily. The samples at pH 6 and 7 presented a pale red color, after 15 days of incubation. At that time, all samples were analyzed by UV-visible spectrometry and the results are shown in Figure 2.

Figure 2 shows the spectra of the samples of albumins incubated for 15 days with HAuCl_4_ in different pH values. The spectra of the solutions at pH ≥ 6 do not present the LMCT band in the UV region. The SPR bands were detected in the spectra of native and NEM-treated HSA and BSA incubated with HAuCl_4_ at pH 6 and 7. The SPR bands were also detected in the spectra of heat-denaturated BSA incubated with HAuCl_4_at pH 6 and 7. For the sample containing heat-denaturated HSA, the SPR band was observed only for the incubation at pH 7. SPR bands were not detected in the spectra of all samples after 15 days of incubation with HAuCl_4_ at pH ≥ 8. The results are summarized in Table 2.

**Table 2:** **Detection of SPR bands for albumin solutions after incubation with HAuCl_4_**.

**pH**	**HSA**			**BSA**		
	**Native**	**NEM-treated**	**Heated**	**Native**	**NEM-treated**	**Heated**
			**85°C**			**85°C**
1–5	−	−	−	−	−	−
6	+	+	+	+	+	−
7	+	+	+	+	+	+
8–12	−	−	−	−	−	−

**Figure 3:**
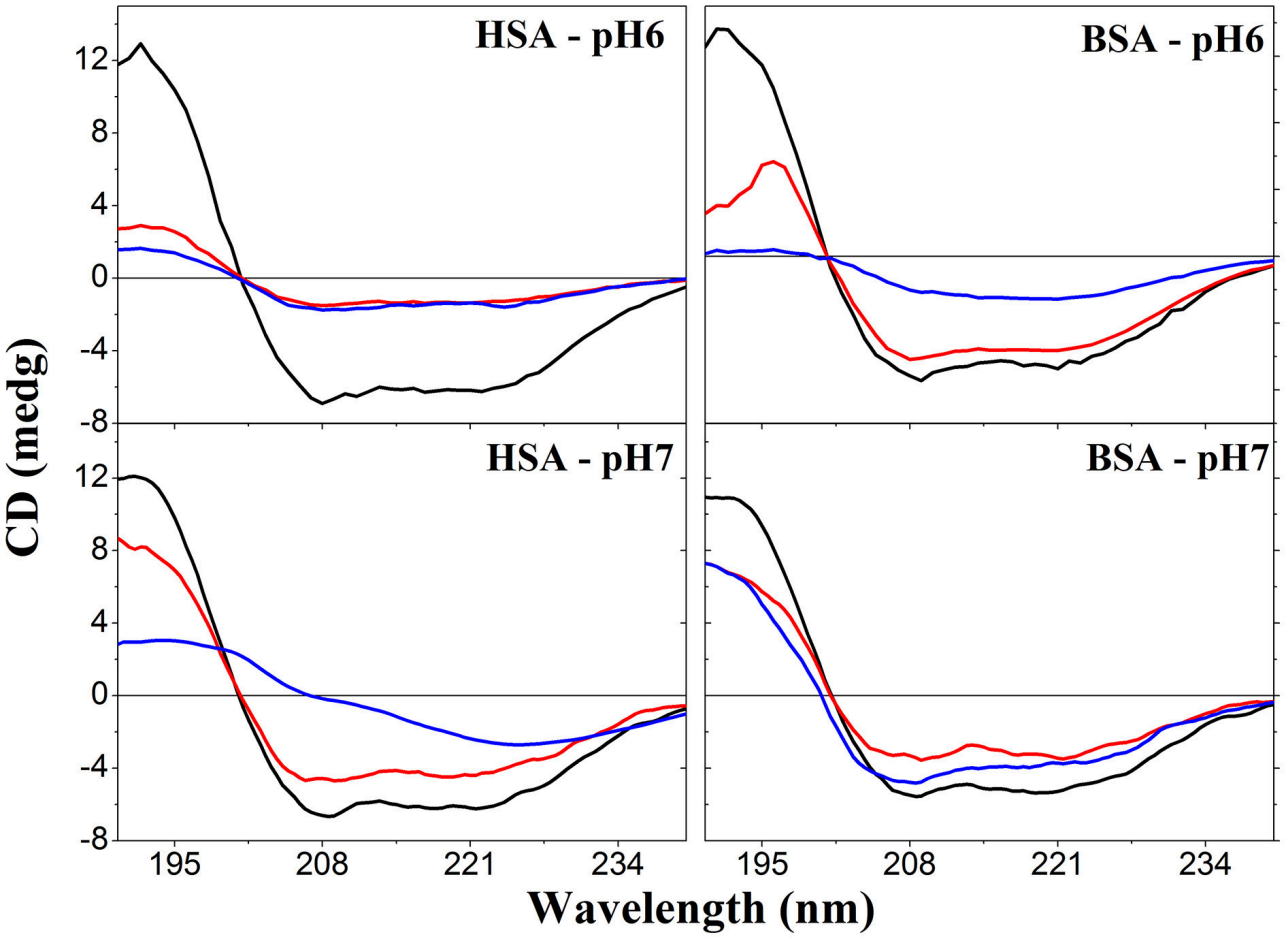
**Far-UV CD spectra of native, NEM-treated and heat-denaturated HSA and BSA**. Upper and lower panels show, respectively, the spectra obtained at pH 6 and 7. For each panel, the black, blue and red lines represent, respectively, the spectra of native, heat-denatured, and NEM-treated albumins.

The formation of GNPs only at pH 6 and 7 suggested that these conditions conjugated favorable conditions of HAuCl_4_ speciation and the reducing capability of albumins. However, the effect of pH on the albumin structure could not be discarded as a condition preventing the formation of GNPs at extreme values of pH. Therefore, it was analyzed the far-UV CD spectra of native, heat-denaturated and NEM-treated HSA and BSA at pH 6 and 7 (Figure 3). Figure 3 shows that the heating and reaction with NEM led to the loss of α-helix content of both albumins. For HSA, the heating and treatment with NEM promoted the most drastic loss of α-helix content. For BSA, heating at pH 6 also promoted a drastic loss of α-helix content. For HSA and BSA at pH 7, the CD spectral features suggest that α-helix content was converted to the random coil and other protein structures. Therefore, possible conformational changes at extreme pH values do not respond to the impairment of GNP formation in these conditions, since the loss of the native structure was also present in albumin samples that were effective for GNP synthesis.

**Figure 4:**
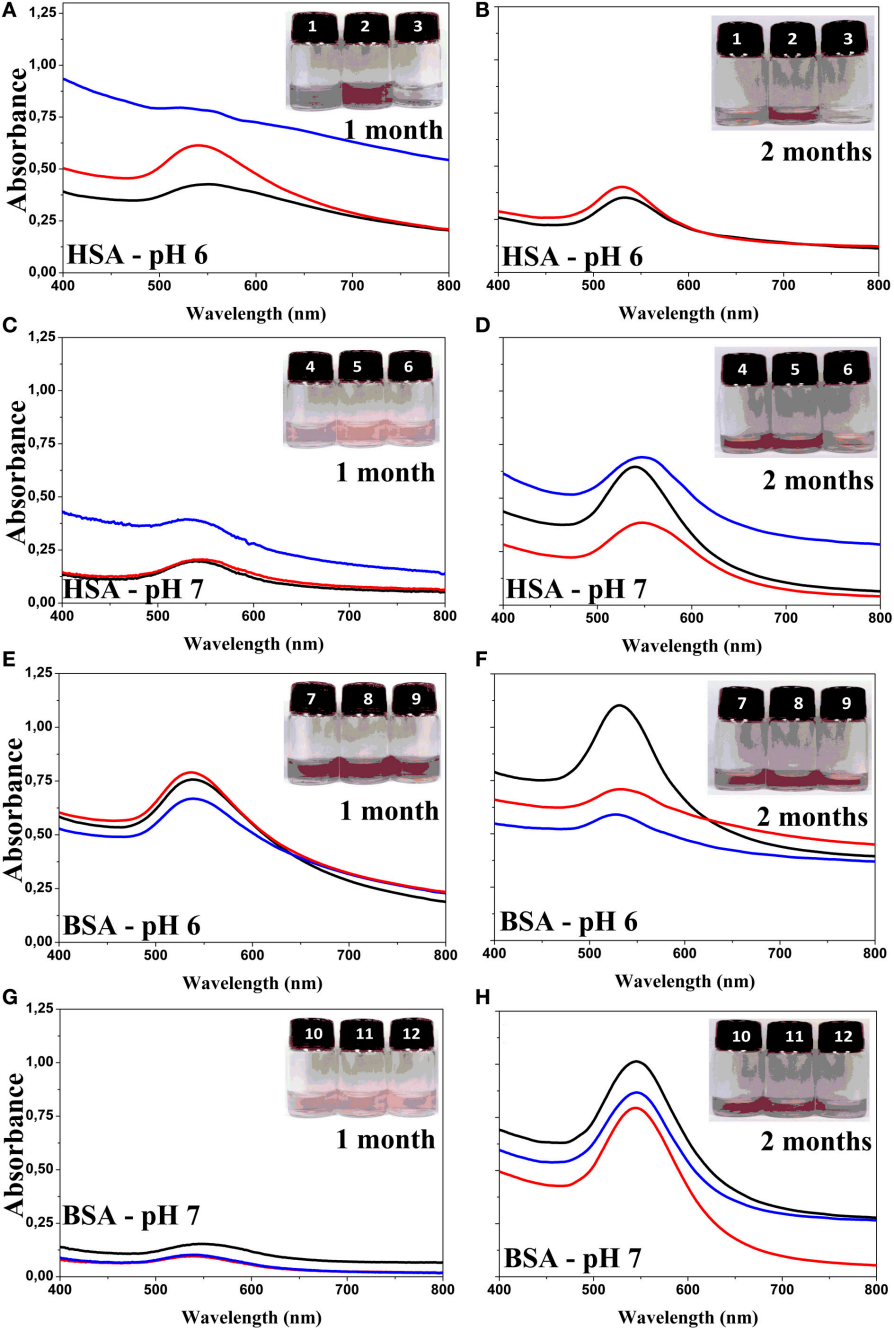
**UV-visible spectra of GNPs obtained using HSA and BSA as reducing and stabilizing agents**. Panels **(A)** and **(B)** show the spectra of GNPs produced with HSA at pH 6.0 after 1 and 2 months of aging, respectively. Panels **(C)** and **(D)** show the spectra of GNPs produced with HSA at pH 7.0 after 1 and 2 months of aging, respectively. Panels **(E)** and **(F)** show the spectra of GNPs produced with BSA at pH 6.0 after 1 and 2 months of aging, respectively. Panels **(G)** and **(H)** show the spectra of GNPs produced with BSA at pH 7.0 after 1 and 2 months of aging, respectively. In each panel, the snap shot of GNP suspension numbered at crescent order are corresponding the spectra represented by black, red, and blue lines, respectively. The black lines correspond to the spectra of the native form of the proteins, red lines to NEM-treated proteins and blue line to heated denaturated proteins. The concentrations of reagents were 0.45 mM of HAuCl_4_, 1.5 μM of serum albumin and 3.0 μM of NEM at pH 6 and 7 as indicated in the panels.

**Figure 5:**
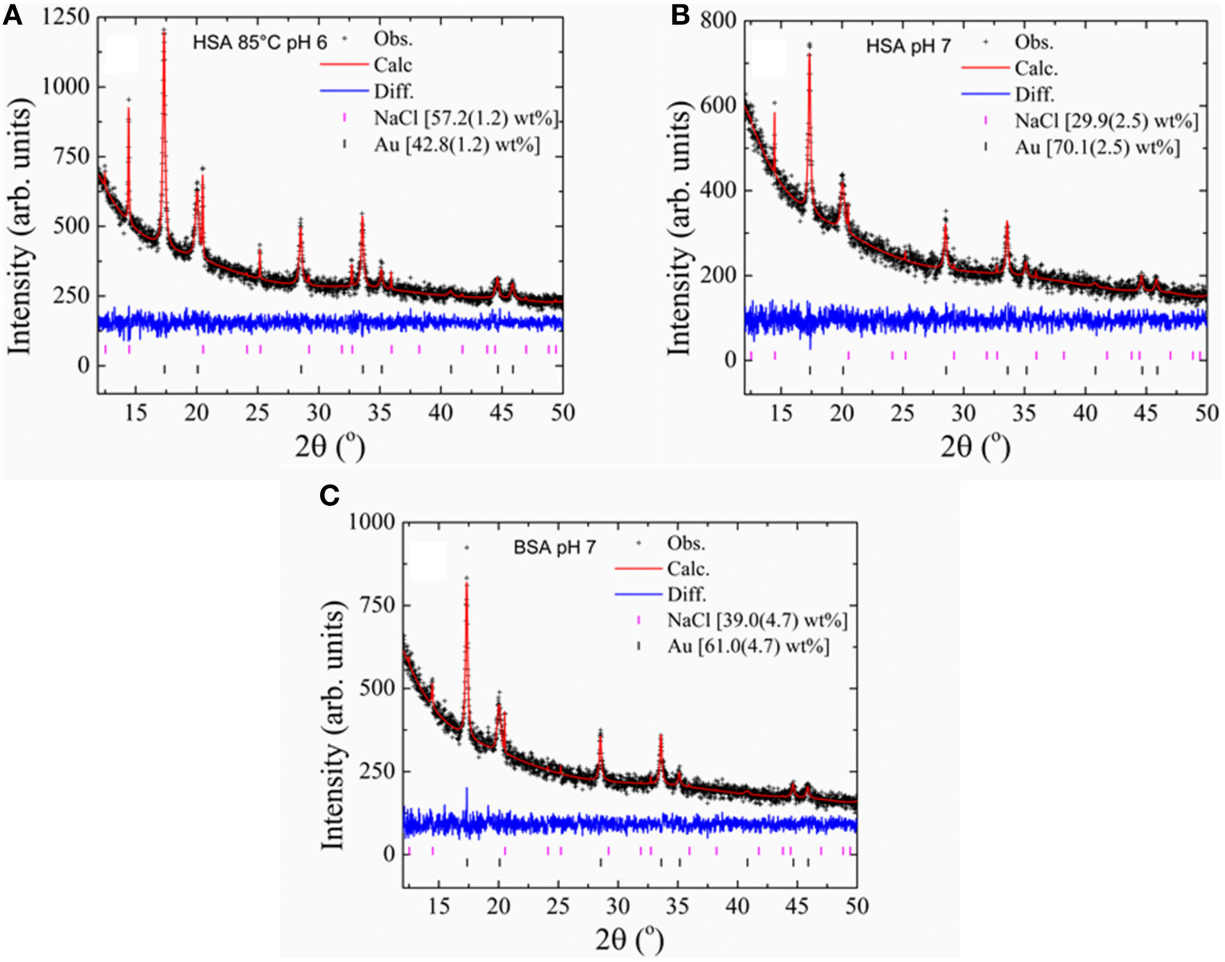
**XRD pattern of GNPs obtained using different forms of albumins**. The data were obtained from the final Rietveld refinement for GNPs obtained with heated HSA at pH 6 **(A)**, native HSA and BSA at pH 7 (**B** and **C**, respectively). Black crosses represent observed data; the continuous red line indicates the calculated pattern and the blue line represents the difference between the observed and calculated patterns. Black and magenta vertical bars at the bottom indicate Bragg reflections for the Au and NaCl phase, respectively.

The UV-visible spectra of the samples incubated at pH 6 and 7 were run again, after 1 and 2 months of incubation (Figure 4). Figure 4 shows the SPR absorption bands of the GNPs produced at pH 6 and 7 using native, heat-denaturated and NEM-treated HSA and BSA. The insets show the snapshot of the GNP suspensions. The SPR absorption bands of GNPs produced using BSA at pH 7 presented similar features and intensities after 1 and 2 months of incubation. The GNPs produced using BSA at pH 6 exhibited increasing intensity after 2 months of incubation. Similar behavior was observed for GNPs produced with HSA at pH 7. GNPs produced using native and NEM-treated HSA at pH 6 presented narrowing of SPR absorption bands without significant changes of the intensity. The GNPs produced using heat-denaturated HSA at pH 6 were not stabilized and precipitated at the bottom of the flasks. Therefore, these GNPs were characterized only by XRD (Figure 5A) using the Rietveld method (Rietveld, 1967, 1969). XRD measurements were also performed for the GNPs produced with native HSA and BSA, at pH 7 (Figures 5B,C, respectively). The XRD patterns clearly show that the gold nanoparticles are crystalline. These data were analyzed according to the Rietveld method by using the program *Topas-Academic* v.5 (Coelho et al., 2011). The samples crystallized in the cubic space group (*Fm* $\bar{3}$ *m*). The presence of NaCl phase resulted from the combination of sodium, the counter ion of the phosphate buffer, with the chloride ion provided by the gold salt. The weight fraction of both NaCl and Au are indicated in the plots in Figure 5, Table 3.

**Table 3:** **Rietveld refinement results for Au phase**.

**NP**	**Average crystallite size (nm)**	**Cell parameter (Å)**	***R_*wp*_*(%)**	***R_*exp*_*(%)**	***R_*Bragg*_*(%)**	**χ^2^(%)**
HAS 85°C pH 6	13.3(3)	4.0672(9)	4.534	5.363	0.510	0.845
Native HAS pH 7	13.7(5)	4.0710(8)	5.187	6.226	0.766	0.833
Native BSA pH 7	14.6(6)	4.0667(8)	5.282	6.120	0.413	0.863
Au (Bulk) 443362	–	4.0796	–	–	–	–

Table 3 presents the results obtained from the Rietveld refinements for the estimated average crystallite size, cell parameters (Å) and the *R*-factors and goodness-of-fit indicators (χ^2^). Table 3 shows that the GNPs have an average crystallite size of 13.8 nm. Also, comparing the unit cell parameters of the GNPs with those of bulk gold (extracted from ICSD card 44362) one can see the latter is ~0.01 Å larger. The GNPs were characterized by a zeta potential (ζ) and dynamic light scattering. Dynamic light scattering method was employed to calculate the sizes of gold nanoparticles coated with serum albumin (hydrodynamic radius–R_H_). The serum albumin coat on gold nanoparticles is expected to cause substantial changes in the ζ potential and hydrodynamic radius of GNPs. Globular proteins such as albumins are enovelated polypeptide chains that bring a pH-dependent net surface charge provided by ionizable lateral chains of the amino acids as well as the carboxy and terminal amino groups. Several forces such as Van der Waals, hydrogen bonds, and solvation forces respond for the covering of GNPs by proteins. Particularly, the thiol groups of the cysteine residues can provide strong interactions of proteins with a GNP. The irreversible and durable binding of proteins on the GNP forms a coat that is known as “hard corona.” Proteins that present faster exchange rates constitute a short-term covering that is known as “soft corona” (Karajanagi et al., 2004; Lundqvist et al., 2008; Saptarshi et al., 2013). The adsorption of proteins on the surface of GNPs is governed by the chemical affinity and the protein tridimensional structure. The latter can limit the ability of the protein to occupy the all GNP surface. The capacity of albumin to form a capping for nanoparticles is important not only for the colloidal stabilization but also for the modulation of the biological effects. As an example, Yan et al. (2013) demonstrated that albumin significantly influences the internalization and biological effects of nanoparticles by cells. In that study, the authors also observed denaturation of albumin upon adsorption onto polymeric nanoparticles. The denatured BSA impaired internalization of the nanoparticles in human monocytic cells, THP-1, compared with the bare particles. Contrarily, the corona provided by unfolded BSA did not affect the uptake of the particles by differentiated macrophage-like cells and triggered phagocytosis mediated by class A scavenger receptor. The analysis of ζ potential and R_H_ provided some information about the efficiency and stability of the GNP corona provided by the albumins (Table 4).

**Table 4:** **Potential (ζ) and Dynamic Light Scattering (DLS)**.

**Reducing agent**	**1 month**	**1 month**		**2 months**	
	**ζ Potential (mV)**	**R_H_ (nm)**	**PDI**	**R_H_ (nm)**	**PDI**
1. Native HSA pH 6	−9 mV (±1)	25.6 nm	0.54	29.8 nm	0.51
2. NEM-treated HSA pH 6^a^	−	24.9 nm	0.56	27.2 nm	0.46
3. Heat-denaturated HSA pH 6^b^	−	−	−	−	−
4. Native HSA pH 7	−11.7 mV (±1)	31.2 nm	0.48	24.1 nm	0.46
5. NEM-treated HSA pH 7^a^	−	34.3 nm	0.47	27.4 nm	0.50
6. Heat-treated HSA pH 7	−27 mV (±1)	31.6 nm	0.37	27 nm	0.42
7. Native BSA pH 6	−4.5 mV (±1)	24.2 nm	0.55	32.9 nm	0.46
8. NEM-treated BSA pH 6	−5 mV (±1)	24.6 nm	0.54	47.2 nm	0.49
9. Heat-treated BSA pH 6^a^	−	27.4 nm	0.54	35.9 nm	0.50
10. Native BSA pH 7	−17 mV (±2)	35.8 nm	0.48	28.3 nm	0.40
11. NEM-treated BSA pH 7^a^	−	34.7 nm	0.46	28.2 nm	0.42
12. Heat-denaturated BSA pH 7	−8 mV (±1)	35.4 nm	0.47	25.8 nm	0.39

^a^ζPotential values did not stabilize.

^b^Measurements impaired by precipitation of the GNPs.

The ζ potential of GNPs produced by HSA and BSA in the native, heat-denatured and NEM-blocked SH forms varied from −5 to −27 mV. These values are consistent with the capping of GNPs by the albumins since the ζ potential of bare GNPs are reported being ~ −38 mV (Slocik et al., 2005). Also, the previous study reported that the ζ potential of GNPs became less negative due to BSA adsorption. The authors attributed the affinity of BSA for the negatively charged GNP surface to electrostatic interactions with the positively charged lysine residues. The variation of ζ potential values could not be securely correlated with different extension of GNP covering because the albumins could interact with GNPs by different sites. The measurements of ζ potential were carried out in ionic strength tenfold lower than that of the synthesis. In this condition, the GNP synthesized using HSA treated with NEM at pH 6 and 7, BSA treated with NEM at pH 7 and BSA heated at 85°C in pH 6 did not stabilize the ζ potential values and registered more negative values progressively. Although stable, the ζ potential of GNP synthesized using HSA heated at 85°C in pH 7 presented the most negative value (−27 mV). Noticeable, these conditions promoted significant loss of HSA and BSA native structure accompanied by thiol blockage or oxidation (Figure 3). The less efficient and durable covering of GNPs by NEM-treated and heat-denaturated albumins at low ionic strength suggests a significant contribution of non-electrostatic interactions of the albumins with GNP surface. The denaturation of proteins exposes hydrophobic groups to the solvent, and these groups could interact with the gold surface. In these conditions, the thiol blockage or oxidation, the latter probably favored upon heating, impaired the formation of a hard corona by thiol coordination with the gold surface. The DLS analysis measured after 1 month of incubation revealed R_H_ values around 25 nm for GNPs synthesized with native and NEM-treated HSA in pH 6. These values were slightly higher (27–29 nm) after 2 months of incubation. GNPs synthesized with heat-denaturated HSA did not become stable impairing the measurement of R_H_ for this sample. The GNPs synthesized with native, heat-denaturated and NEM-treated HSA in pH 7 presented R_H_ values around 32 nm. This value decreased to ~26 nm after 2 months of incubation. Similar behavior was observed for the GNPs synthesized with the BSA samples at pH 7, and opposite behavior was observed for the GNPs synthesized with BSA samples at pH 6. The temporal increase of the R_H_ values could be assigned to the increase of GNP capping by the albumins. Otherwise, the temporal decrease of R_H_ values could be assigned to a partial loss of the GNP capping by albumins.

**Figure 6:**
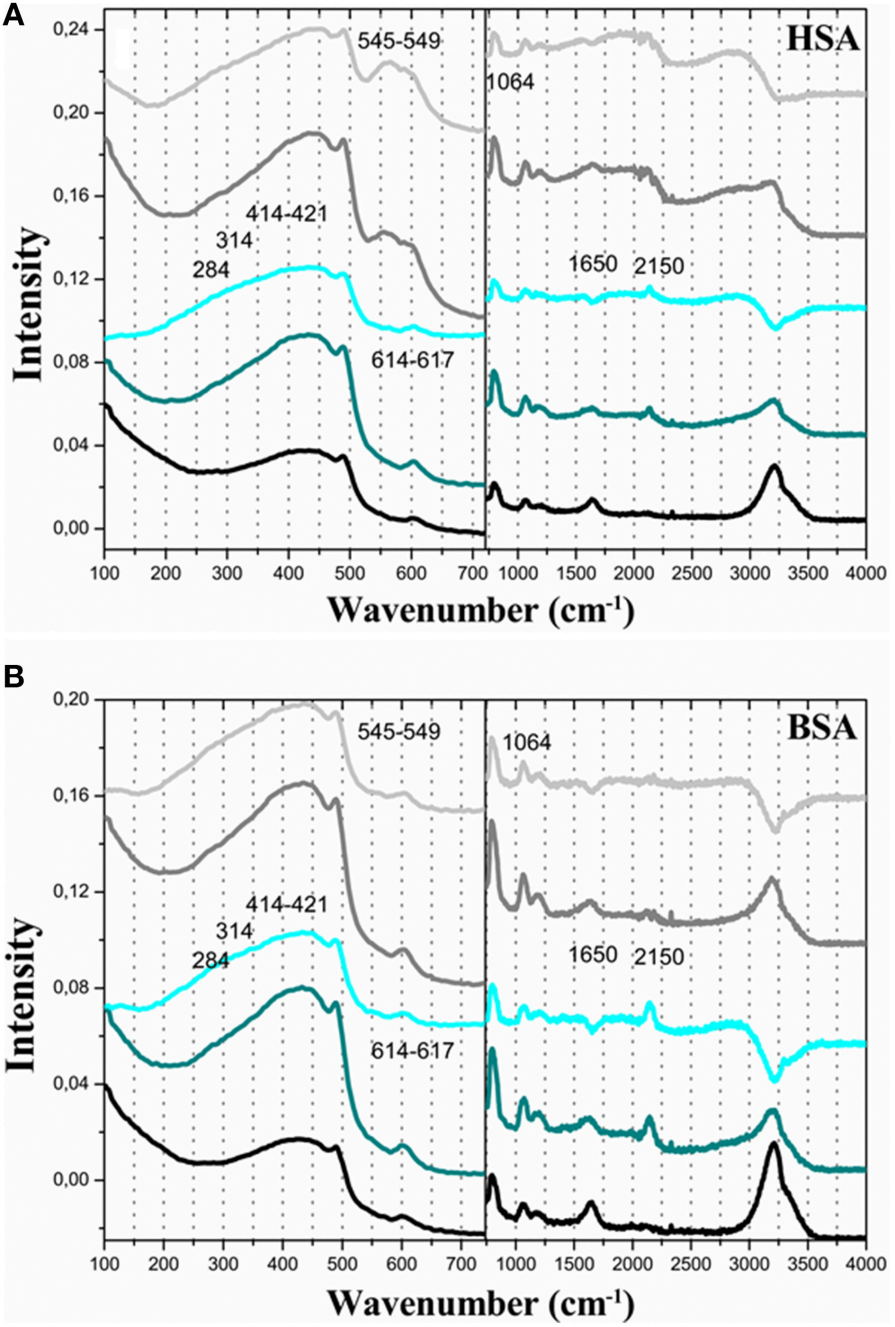
**FT-RAMAN spectra of GNP obtained using human and bovine serum albumin with the agent for reducing aqueous**. The different human **(A)** and bovine **(B)** serum albumin (line black corresponds native form, line red corresponds NEM and line blue corresponds denaturation by temperature. The concentrations of HAuCl_4_ 0.45 mM, serum albumin 1.5 μM, and NEM 3.0 μM. All solution was prepared at pH 7.

The suspensions of GNP synthesized with native albumins were analyzed by Raman spectrometry to get information about the type of interaction involved in the capping of the GNPs by the albumins. Figures 6A,B show the Raman spectra of the GNPs synthesized and capped with native HSA and BSA, respectively. In Figures 6A,B, the black lines correspond to the spectra of HSA and BSA solutions. The dark cyan lines correspond to the spectra of albumins associated to GNPs synthesized at pH 6. The cyan lines correspond to the differential spectra obtained by subtracting the control spectra of the protein solution from the spectra obtained at pH 6. The gray lines correspond to the spectra of albumins associated to GNPs synthesized at pH 7. The light gray lines correspond to the differential spectra obtained by subtracting the control spectra from the spectra obtained at pH 7. In Figures 6A,B, the differential spectra show the loss of the band around 1650 cm^−1^. In the Raman spectrum of proteins the band amide I, around 1650 cm^−1^, is assigned to the contribution of the α-helix content. The loss of the 1650 cm^−1^ band is consistent with denaturation of the albumins after association with the gold surface (Slocik et al., 2005). The differential spectra show a signal increase and additional broadening of the band in the spectral range from 200 to 550 cm^−1^. This broadband involves overlapped contributions of CSH bending mode (β_*CSH*_) in the region of 284–315 cm^−1^ and C-S stretching mode (ν_*CS*_) in the region of 414–421 cm^−1^. Therefore, a SERS (surface enhanced Raman spectroscopy) effect was observed for these vibrational modes of the albumins associated with GNP surface. SERS effect was also observed for the band peaking at 601 cm^−1^ that could be a blue shifted contribution of δ_CSS_ and ν_CSS_ modes. The SERS effect was observed for the bands with peaks at 786 (HSA) and 802 (BSA) that are assigned to the lateral chain of tryptophan and also for the band peaking at 1071 cm^−1^ that is a contribution of C-C and C-S asymmetric stretching (Fontana et al., 2013). Noticeable is the appearance of a band at 2150 cm^−1^ for the albumins capping GNPs at pH 6. This band is assigned to the coordination of protonated SH with the gold surface (Bandyopadhyay et al., 2015). The absence of this contribution in the spectra of the GNPs synthesized at pH 7 is consistent with the lowered p*K*_*a*_ of the SH group in the albumin structure that makes this group predominantly deprotonated at pH 7.

## Discussion

**Figure 7:**
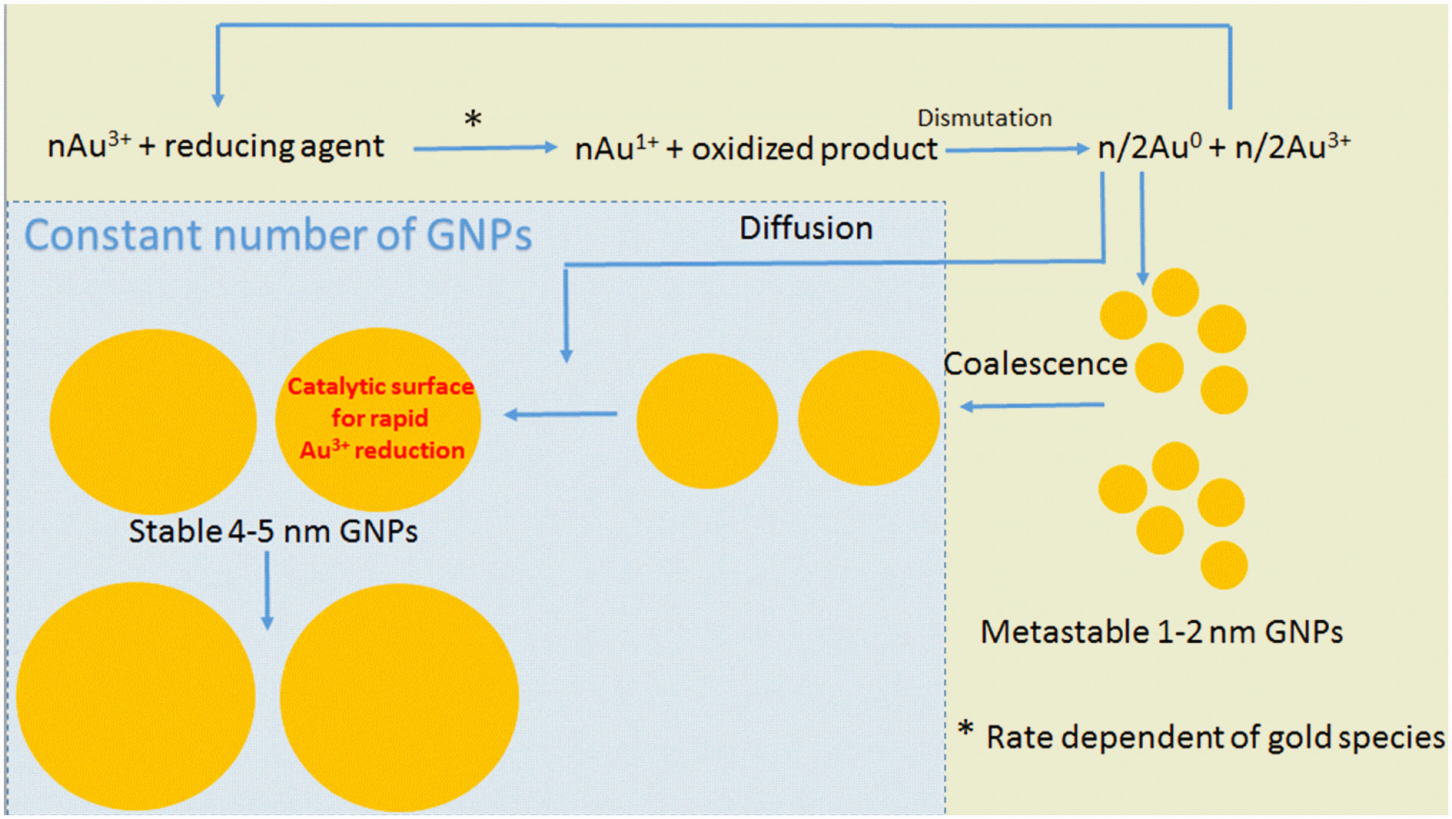
**Schematic representation of the mechanism of GNP synthesis using mild reducing agents**. The steps showed with yellow background result in increasing number of particles, and the steps showed with blue background maintain the number of particles constant.

**Figure 8:**
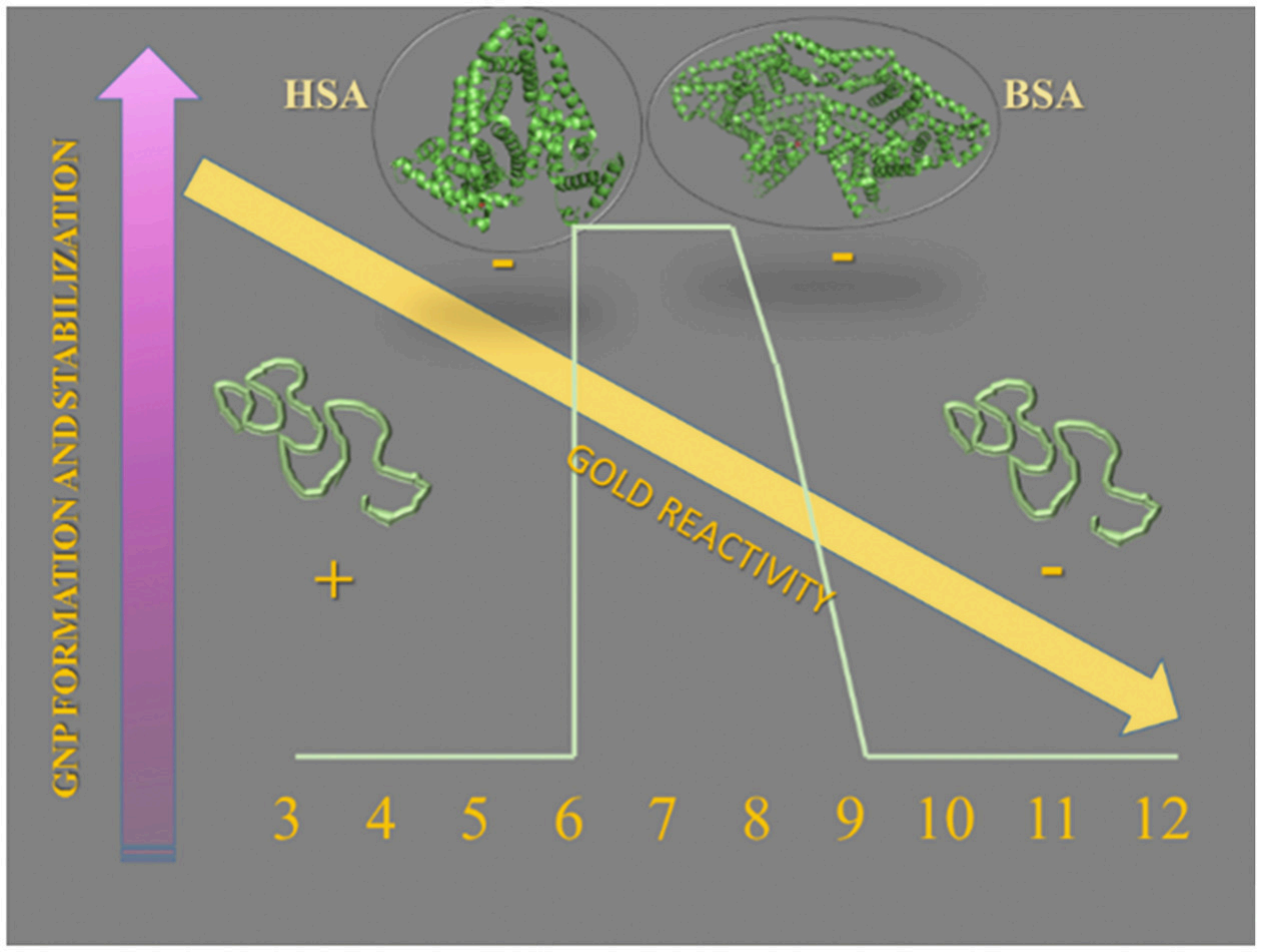
**Schematic representation of the conditions that influences the synthesis of GNPs using HSA and BSA as reducing and stabilizing agents**. At the optimal pH range, HSA and BSA are in the native conformation and are represented by images created in PyMol program loaded with PDB files. At the acid pH range, below pH 5, the albumins are positively charged and above the pI (4.7), the albumins are negatively charged.

A large number of studies have been devoted to the characterization, stabilization and different applications of the metal nanoparticles (MNPs). However, there are relatively few scientific contributions concerned with the mechanisms of MNP formation. A detailed discussion of the mechanisms of nucleation and growth of nanoparticles can be found in the review of Thanh et al. (2014). The chemical synthesis of MNPs is a bottom-up process that involves three general steps: reduction of the metal ion, formation of metal clusters (nucleation) and growth to form a final stable MNP with a specific form and size. The general mechanism of nucleation and growth of NPs proposed by LaMer involves three steps (LaMer and Dinegar, 1950; LaMer, 1952; Thanh et al., 2014). The first step involves a fast increase of monomer concentration in solution, which in the case of MNP synthesis refers to the formation of metal atoms from the reduction of the corresponding ions. The second step involves a burst-nucleation of monomers leading to a significant decrease in the monomer concentration in solution. The low concentration of monomers after the burst-nucleation step impairs the occurrence of significant nucleation after this point. In the third step, the particles grow in a diffusion-controlled process. The Finke-Watzky mechanism involves simultaneous nucleation and growth. According to this mechanism, the nucleation is a slow continuous process, and the surface growth is an autocatalytic process, which is not

diffusion controlled (Thanh et al., 2014). Different mechanisms for NP growth have been proposed: Ostwald ripening, digestive ripening, coalescence, orientated attachment and intraparticle growth. Ostwald ripening involves the re-dissolution of smaller and less stable NPs providing monomers for the additional growing of larger NPs. Digestive ripening is an inverse process in which smaller NPs grow using monomers provided by the re-dissolution of larger NPs. Coalescence involves a random joining of seeds and oriented attachment involves the growing in a common crystallographic alignment. The latter mechanism forms continuous crystallographic planes. The intraparticle growth is a mechanism that modifies the NP shape by the diffusion of metal atoms along the particle surface. Successive growth mechanisms can operate during the formation of MNPs. For the synthesis of GNPs, even using sodium citrate (Turkevitch method), the pH-dependent speciation of gold ion influences the kinetics and mechanisms. The synthesis of GNPs at the pH range 3.7–6.5 has [AuCl_3_(OH)]^−^ as the intermediate. In this conditions, it was observed a LaMer burst nucleation (10 s), followed by rapid coalescence of seeds with subsequent intraparticle ripening. The GNP synthesis using sodium citrate at a higher pH range (6.5−7.7), having [AuCl_2_(OH)]^2−^ and [AuCl(OH)]^3−^ as the intermediates, exhibited a longer nucleation process (~60 s) and slower growth. The majority of the synthetic procedures uses mild reducing agents such as citrate. In this condition, the chemical reaction directs the kinetics of the MNP growth. Therefore, the synthesis of MNPs using mild reducing agents involves a four-step model of synthesis (Polte et al., 2010a,b). The four steps were derived directly from experimental results obtained by UV-vis, SAXS, XANES, SEM, and TEM (Polte, 2015). Figure 7 depicts the steps of GNP synthesis using mild reducing agents. According to Figure 7, the formation of GNPs using mild reducing agents involves the reduction of Au^3+^ to Au^1+^ followed by dismutation of Au^1+^ to Au^0^ and Au^3+^. The gold atoms aggregate to form metastable seeds of 1–2 nm that coalesce to form larger GNPs. From this step onwards the number of particles remains constant, and the GNPs grow by the association of gold atoms formed by subsequent reduction of Au^3+^. When the particles attain diameters of 4–5 nm, it is observed a rapid increase in the gold reduction rate and GNP growing. The increase of gold reduction rate in this step is assigned to the autocatalytic surface provided by the GNPs. In this mechanism, the rate of the first step, i.e., the Au^3+^ reduction, is pH-dependent. HAuCl_4_ suffers pH-dependent hydrolysis that leads to the speciation of aqueous HAuCl_4_. On the other hand, the steps of GNP synthesis by mild reducing agents were determined using low molecular weight agents such as sodium citrate. Although citrate and its oxidation products are efficient capping and stabilizing agents for the GNPs (Mpourmpakis and Vlachos, 2008; Jiang et al., 2010; Toma et al., 2010; Leng et al., 2015), these low molecular weight molecules do not provide a template and different microenvironments for directing the NP growing. Therefore, the synthesis of GNPs using proteins as reducing agents—the biomineralization—presents also a higher degree of complexity. Recently, it was investigated the early steps of protein-directed GNP synthesis by the temporal analysis of the process in a single crystal of lysozyme (Wei et al., 2011). In this study, the step of reduction of Au^3+^ to Au^1+^ was skipped by promoting the crystallization of lysozyme in the presence of Au^1+^. In the first day, two gold atoms were identified in the lysozyme crystal structure. One Au^+1^ was found coordinated with the 1-N of His15 and another gold ion, probably Au^3+^ was coordinated with Tyr23. The number of gold atoms coordinated with the protein increased concomitantly with the crystal growth up to the number of eight gold atoms on the 90th day of analysis. This study exemplifies that the growth of GNPs directed by macromolecules such as proteins is a more complex process that is influenced by the molecular tridimensional structure and accessibility to coordinating groups. Therefore, in the present study, we combined the variable pH-dependent gold speciation with albumin structure modulated by pH, heating and SH blockage by NEM. The use of NEM-treated albumin also provided information about the importance of the protein thiol group for the GNP synthesis. At the pH range, 3–5 albumins exhibit significant loss of secondary and tertiary structure (Dockal et al., 2000). However, the acid-induced loss of albumin secondary structure is less drastic than that promoted by guanidine. It is also important to consider that the pI of albumins is 4.7, and consequently, the liquid charge of albumin at the pH range 3–5 varies from positive to zero and slightly negative values. However, although, the speciation of gold at the pH range 3–5 provided the most reactive species, the liquid charge, and structure of albumin did not favor the formation of GNPs. At pH 8, the formation of GNPs as indicated by the appearance of a red color in the solution was observed only after around 2 months of incubation. In this condition, albumins retain the native structure, and the slower rate of GNP formation might be assigned to the predominance of less reactive species of gold ions. At the pH range 9–10, domains I and III of albumins lose more significantly the native structure (Ahmad et al., 2004). In these conditions, GNP formation was not observed. At the pH range 11–12, the native structure of domain II of the albumins is also affected. In these conditions, it was observed the formation of large black aggregates at the bottom of the flasks, after 2 months of incubation. The most favorable conditions that were observed for the formation and stabilization of GNPs by HSA and BSA were pH 6 and 7 for the proteins in the native form. These conditions are the unique that combine native structure and net negative charge of the albumins with significant concentrations of the most reactive gold species. At pH 6 and 7, the loss of HSA and BSA native structure promoted by heat and NEM treatment did not prevent GNP formation but affected the colloidal stability. As previously evidenced by studies with recombinant albumin, Cys34 of albumins are not crucial for gold ion reduction. However, the lower stability and loss of capping of GNP produced using albumins with the thiol group blocked by NEM or probably oxidized by heating suggest that Cys34 can contribute to the GNP stabilization. The principal findings of the present study are summarized in Figure 8.

## Author contributions

EM designed experiments, synthesized the gold nanoparticles, and analyzed the samples by UV-visible spectrometry and zeta potential. She also contributed with artwork of the figures and manuscript writing. AT synthesized the gold nanoparticles and assisted with manuscript writing. AB, JA, and DL contributed with the preparation of reagents and circular dichroism measurements. CD, LA, and FG provided the measurements and analysis of zeta potential and hydrodynamic radius. FC and FF contributed with the XRD measurements and analysis. IN is the corresponding author, coordinated the project and contributed to the writing of the manuscript.

### Conflict of interest statement

The authors declare that the research was conducted in the absence of any commercial or financial relationships that could be construed as a potential conflict of interest.
